# The Use of Social Media in Detecting Drug Safety–Related New Black Box Warnings, Labeling Changes, or Withdrawals: Scoping Review

**DOI:** 10.2196/30137

**Published:** 2021-06-28

**Authors:** Jae-Young Lee, Yae-Seul Lee, Dong Hyun Kim, Han Sol Lee, Bo Ram Yang, Myeong Gyu Kim

**Affiliations:** 1 College of Pharmacy Chungnam National University Daejeon Republic of Korea; 2 College of Pharmacy Ewha Womans University Seoul Republic of Korea; 3 Graduate School of Pharmaceutical Sciences Ewha Womans University Seoul Republic of Korea

**Keywords:** adverse event, black box warning, detect, pharmacovigilance, real-world data, review, safety, social media, withdrawal of approval

## Abstract

**Background:**

Social media has become a new source for obtaining real-world data on adverse drug reactions. Many studies have investigated the use of social media to detect early signals of adverse drug reactions. However, the trustworthiness of signals derived from social media is questionable. To confirm this, a confirmatory study with a positive control (eg, new black box warnings, labeling changes, or withdrawals) is required.

**Objective:**

This study aimed to evaluate the use of social media in detecting new black box warnings, labeling changes, or withdrawals in advance.

**Methods:**

This scoping review adhered to the Preferred Reporting Items for Systematic reviews and Meta-Analyses extension for Scoping Reviews checklist. A researcher searched PubMed and EMBASE in January 2021. Original studies analyzing black box warnings, labeling changes, or withdrawals from social media were selected, and the results of the studies were summarized.

**Results:**

A total of 14 studies were included in this scoping review. Most studies (8/14, 57.1%%) collected data from a single source, and 10 (71.4%) used specialized health care social networks and forums. The analytical methods used in these studies varied considerably. Three studies (21.4%) manually annotated posts, while 5 (35.7%) adopted machine learning algorithms. Nine studies (64.2%) concluded that social media could detect signals 3 months to 9 years before action from regulatory authorities. Most of these studies (8/9, 88.9%) were conducted on specialized health care social networks and forums. On the contrary, 5 (35.7%) studies yielded modest or negative results. Of these, 2 (40%) used generic social networking sites, 2 (40%) used specialized health care networks and forums, and 1 (20%) used both generic social networking sites and specialized health care social networks and forums. The most recently published study recommends not using social media for pharmacovigilance. Several challenges remain in using social media for pharmacovigilance regarding coverage, data quality, and analytic processing.

**Conclusions:**

Social media, along with conventional pharmacovigilance measures, can be used to detect signals associated with new black box warnings, labeling changes, or withdrawals. Several challenges remain; however, social media will be useful for signal detection of frequently mentioned drugs in specialized health care social networks and forums. Further studies are required to advance natural language processing and mine real-world data on social media.

## Introduction

Clinical trials are a primary measure of the efficacy and safety of drugs before they are marketed. However, the limited number of subjects and study period makes it difficult to detect rare adverse drug reactions (ADRs) [1]. For example, 10,000 subjects are required to detect a very rare ADR (with a frequency of <1 in 10,000 individuals). More subjects are needed to obtain significant results. Furthermore, the controlled environment of clinical trials does not fully reflect the effects of different ages, comorbidities, and drug-drug interactions on ADRs in the real world. Hence, it is important to investigate the real-world data (RWD) of marketed drugs for mining ADR signals. Pharmacovigilance is defined as the science of detecting, assessing, understanding, and preventing drug-related adverse effects or problems [2]. Postmarketing surveillance studies and mining of spontaneous adverse event reporting systems are the 2 principal methods of pharmacovigilance.

In recent years, social media has become an essential part of everyday life. Social media platforms (eg, Facebook, Twitter, and patient forums) are where people share experiences and opinions. Patients use social media to increase their health knowledge and exchange advice and information [3]. Social media has become a new source of RWD on ADRs. Researchers expect social media to identify signals that conventional pharmacovigilance methods (eg, postmarketing surveillance studies and spontaneous adverse event reporting systems) have not identified or to identify signals earlier than conventional methods. In addition, social media can be monitored in real time, and adverse events caused by off-label use, unknown in clinical trials, can be detected.

Many studies have investigated the detection of early ADR signals on social media. Tricco et al [4] conducted a scoping review, which included 77 pharmacovigilance studies that used social media, from 2001 to 2016. Another review by Pappa and Stergioulas [5] qualitatively analyzed 100 pharmacovigilance studies that used social media, from 2007 to 2018. Both these reviews summarized the social media platforms used in research and data analysis methods, and the advantages and challenges of pharmacovigilance using social media. Compared to conventional pharmacovigilance methods, pharmacovigilance studies using social media are still in their infancy [4,5]. However, social media has the potential to complement existing pharmacovigilance systems [6].

The reliability of signals derived from social media is questionable [7]. Positive controls are essential for ensuring signal accuracy and verifying the usefulness of social media for mining previously unknown ADRs. Considering the purpose of pharmacovigilance, new black box warnings, labeling changes, and withdrawals are appropriate as positive controls, rather than well-known ADRs already on the drug label. Patients usually post mild and common ADRs (eg, pain or fatigue) on social media platforms; hence, testing whether mining social media data can detect significant ADRs (appearing as new black box warnings, labeling changes, or withdrawals) is necessary [8].

This scoping review aimed to determine how well social media helps detect new black box warnings, labeling changes, or withdrawals in advance.

## Methods

### Search Strategy

This study followed a preplanned protocol and adhered to the Preferred Reporting Items for Systematic reviews and Meta-Analyses extension for Scoping Reviews checklist [9]. Two biomedical databases, PubMed and EMBASE, were searched to identify relevant literature. Search terms (Multimedia Appendix 1) consisted of 15 popular social networks worldwide and sites mentioned in previous scoping reviews [4,10]. There were no restrictions on publication language and publication year. The search was conducted on January 7, 2021. We searched the reference lists of relevant literature (eg, recent reviews and selected articles in this scoping review) for inclusion. Additional Google Scholar searches have been made to include grey literature. EndNote X9 (Clarivate Analytics) was used to manage the literature.

### Study Selection

Two authors independently evaluated the eligibility of the studies. Articles were included if they focused on the analysis of drug safety–related new black box warnings, labeling changes, or withdrawals from social media data. Studies were excluded if they did not study new black box warnings, labeling changes, or drug withdrawals, did not use social media data (eg, data of the US Food and Drug Administration [FDA] Adverse Event Reporting System [FAERS]), or were not original articles (eg, reviews, protocols, and conference abstracts). If the same data were assessed using the same analytic method, an article that could encompass other studies was selected.

Owing to the nature of computer science research, conference proceedings that contained data were included as an exception. Unlike most other academic disciplines, computer science often considers conference proceedings as a last-resort means of reporting research findings [11]. Conference proceedings are typically between 4000 and 7000 words in length, similar to journal articles, which allows sufficient details of the study to be reported [11].

Study selection proceeded in 2 stages. First, titles and abstracts were screened for eligibility. Second, full texts were reviewed to finalize the articles for analysis. Any discrepancy between the 2 authors was resolved through discussion.

### Data Extraction

Data were extracted by an author and recorded in a preprepared data extraction table. The extracted data were reviewed by other authors, and the data extraction form was continuously modified. The extracted data included article characteristics (eg, author and publication year), target social media platforms, study drugs, analytical methods, main results, and limitations mentioned by the authors.

### Results Synthesis

The study results were aggregated or summarized during qualitative synthesis. Additionally, descriptive statistical analysis was conducted for the frequency and proportion of the social media platforms and analytical methods used in the studies. Social media platforms were categorized in accordance with the classification of Pappa and Stergioulas [5]: generic social networking sites (SNS) and specialized health care social networks and forums. Generic SNS include Facebook and Twitter, and specialized health care social networks and forums include generic health-centered SNS (eg, PatientsLikeMe, DailyStrength, MedHelp, and WebMD), medicine-focused sharing platforms (eg, Ask a Patient and Medications.com), and disease-specific web-based health forums.

## Results

### Characteristics of Selected Studies

**Figure 1:**
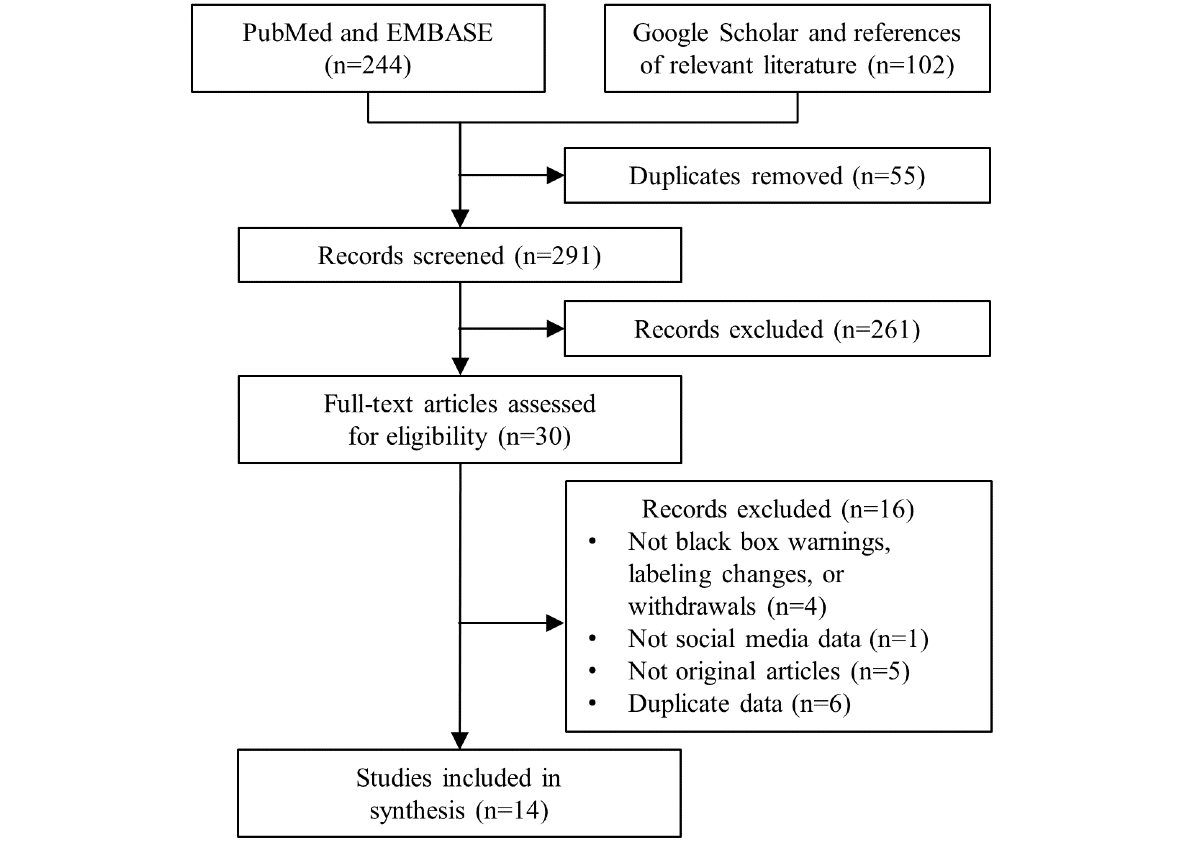
Flowchart of the protocol of the scoping review.

Figure 1 shows the study selection process. We identified 75 articles from PubMed and 169 from EMBASE. Google Scholar and bibliographic searches yielded 87 and 15 studies, respectively. Through the deduplication and 2-step selection process, 14 studies were finally included in this scoping review [12-25]. Table 1 provides the details of the included studies and the social media sources used in each study.

**Table 1:** Characteristics of social media sources.

Number	Author (year)	Social media platforms	Duration	Study drugs	Number of posts
1	Caster et al (2018) [12]	Twitter, Facebook, and 407 patient forums	March 2012-March 2015	75 drugs (Harpaz et al [26] reference set and WEB-RADR reference set)	6,279,424 posts from Twitter and Facebook (690,492 posts with an indicator score threshold of 0.4); 42,721 posts from 407 patient forums using with an indicator score threshold of 0.7
2	Pierce et al (2017) [13]	Facebook and Twitter	March 2009-October 2014	10 drugs	935,246 posts (704,283 nonspam posts)
3	Duh et al (2016) [14]	Ask a Patient	2001-2014	Sibutramine and atorvastatin	270 posts on sibutramine and 998 posts on atorvastatin
4^a^	Yang et al (2015) [15]	MedHelp	1997-2011	20 drugs with >500 threads for each	16,344 posts (8053 posts on 10 drugs that were on alert or had a revised label)
5^a^	Yang et al (2015) [16]	MedHelp	1997-2011	20 drugs with >500 threads for each	16,344 posts
6	Feldman et al (2015) [17]	MedHelp, exchanges.webmd.com, HealthBoards, and ehealthforum.com	1999-2013	Cholesterol-lowering drugs and antidepressants	41,086 posts for cholesterol-lowering drugs and 273,990 for antidepressants
7	Coloma et al (2015) [18]	Facebook, Google+, and Twitter	Until September 2014	Rosiglitazone	2537 posts related to rosiglitazone and cardiovascular events
8	Patki et al (2014) [19]	DailyStrength	—^b^	20 normal and 18 black box drugs^c^	20,486 posts (normal: 10,399, black box: 7327, withdrawn: 2760)
9	Abou Tamm et al (2014) [20]	Three French websites (Doctissimo, Atoute.org, and Vivelesrondes)	—	Benfluorex	220 initial posts and 660 secondary posts
10	Adjeroh et al (2014) [21]	Twitter and general web search queries	2008-2012	46 drugs that had a Food and Drug Administration alert	2 million posts on Twitter
11	Wu et al (2013) [22]	Online discussions using forum search engines such as Google Discussion Search	2000-2011	4 drugs	178,871 posts
12	Liu et al (2013) [23]	Diabetes patient forum	February 2009-November 2012	Antidiabetic drugs	185,874 posts
13	Chee et al (2011) [24]	Yahoo Groups	—	Not prespecified	Not mentioned^d^
14	Chee et al (2009) [25]	Yahoo Groups	—	Natalizumab, rofecoxib, and celecoxib	20,000 posts on natalizumab and 867,659 posts on rofecoxib and celecoxib

^a^Same data but different analytical methods were used.

^b^—: data not available.

^c^The number of drugs withdrawn was not mentioned.

^d^There is a total of 12,519,807 messages in 27,290 public Health & Wellness Yahoo! groups.

**Figure 2:**
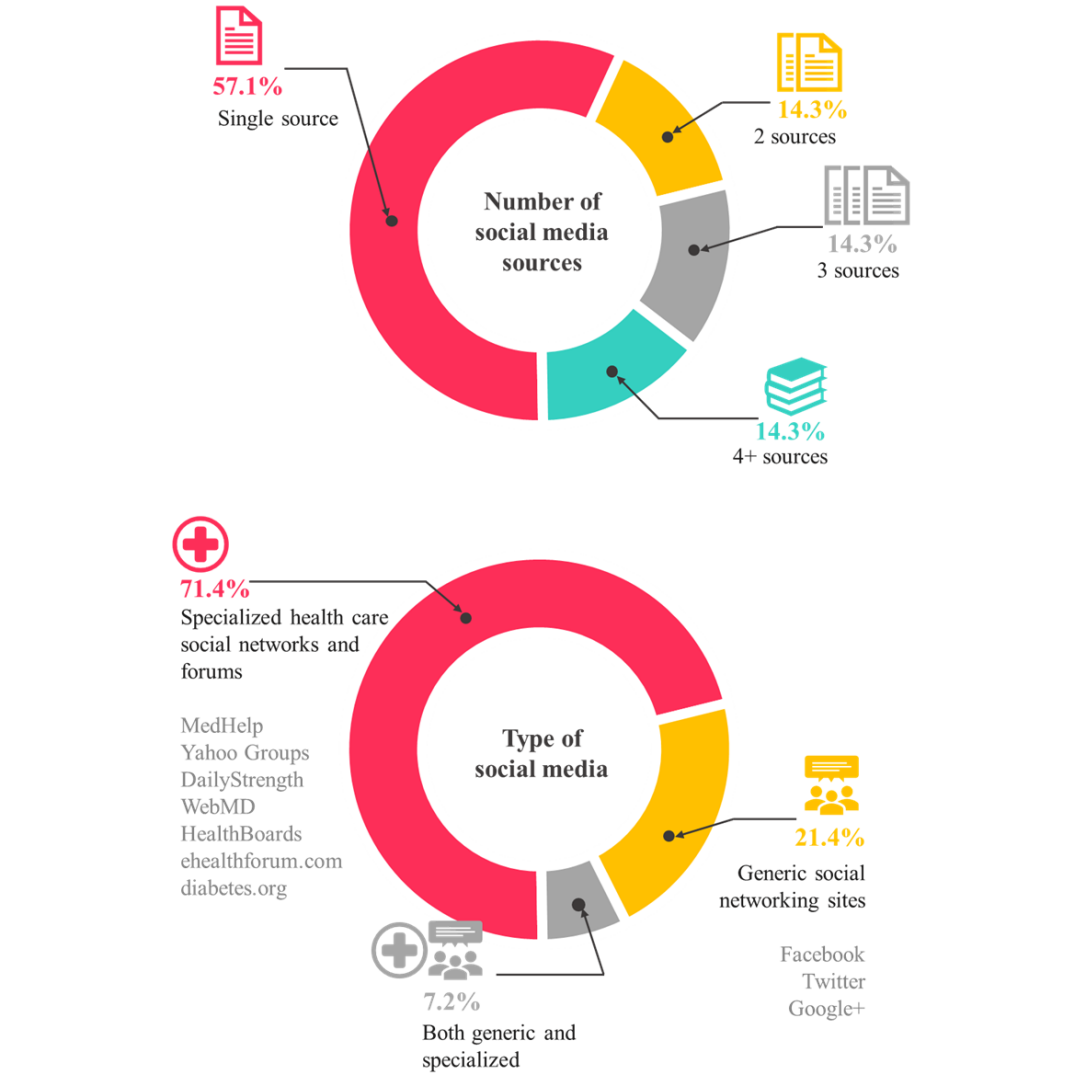
Number and type of social media sources.

Figure 2 shows the number and type of social media sources. Most studies (n=8 of 14, 57.1%) collected data from a single source. Meanwhile, Caster et al [12] collected posts from Twitter, Facebook, and 407 patient forums. Specialized health care social networks and forums were used in 10 (71.4%) studies, and 3 (21.4%) studies used generic SNS. MedHelp (n=4 of 14, 28.6%), Twitter (n=4 of 14, 28.6%), Facebook (n=3 of 14, 21.4%), and Google (n=3 of 14, 21.4%) were frequently used sites.

The number of posts collected varied from hundreds to tens of millions according to the type of sources and study drugs (Table 1). Studies using generic SNS platforms collected a relatively large number of posts compared to those using specialized health care social networks and forums. However, the large volume of posts collected on generic SNS are noisy and difficult to process [5]. For example, Pierce et al [13] sampled 935,246 posts that named 10 selected drugs from Facebook and Twitter over 5.5 years. Among them, 98,252 posts resembled adverse events (Proto-AEs), and only 6 posts described certain, probable, and possible cases of interest [13]. Yang et al [15] collected 8053 posts that named 10 drugs over 4 years on MedHelp.

### Methods Used for Detecting New Black Box Warnings, Labeling Changes, or Withdrawals

**Figure 3:**
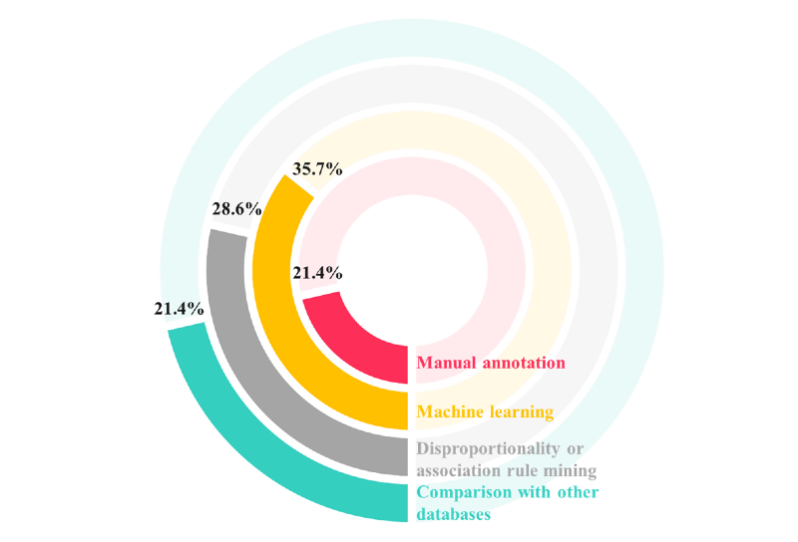
Analytical methods in the studies included in this scoping review.

The analytical methods used in the studies varied considerably. Figure 3 indicates the analytical methods used in each study (Multimedia Appendix 1). Three (21.4%) studies conducted by Duh et al [14], Coloma et al [18], and Abou Taam et al [20] manually annotated posts for the presence or absence of selected adverse events. After manual annotation, a time-series analysis or frequency analysis was conducted. This analysis was possible because the number of posts used in these 3 studies ranged from hundreds to thousands. However, this analytical method is difficult to implement with a higher number of posts.

Five (35.7%) studies adopted machine learning algorithms for detecting ADRs from social media. Feldman et al [17] utilized an unsupervised relation extraction for mining drug-ADR relations. Patki et al [19] used machine learning algorithms for the binary classification of social media posts as ADR or non-ADR. Liu et al [23] used a kernel-based learning method to extract adverse events in patient forums and then used semisupervised learning algorithms to classify report sources into patients’ experiences or not. Chee et al [24] used machine learning algorithms to classify drugs as watchlist and nonwatchlist drugs. Pierce et al [13] used automated classifiers that identified Proto-AEs and then manually reviewed the cases and assessed causality.

Caster et al [12] conducted a disproportionality analysis to detect signals and compared the area under the receiver operating characteristic curve with a World Health Organization global pharmacovigilance database (VigiBase database). Yang et al [15], Yang et al [16], and Feldman et al [17] computed a lift measure for association rule mining to evaluate the likelihood of a particular drug-ADR relationship. Other methods include a tensor-based technique [16], discriminative classification and generative modeling [22], sentiment analysis [25], and peak-labeling signal fusion [21].

### Detection Performance

**Figure 4:**
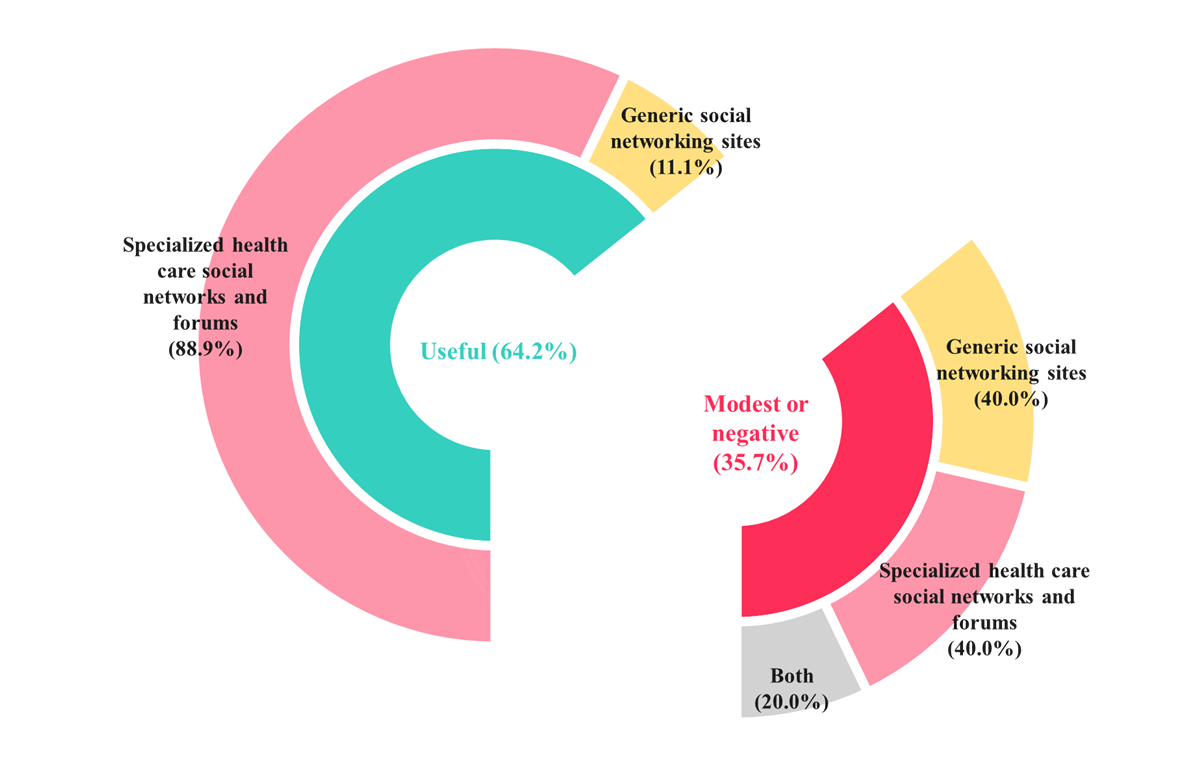
Perspectives on pharmacovigilance using social media and sources of social media.

Nine (64.2%) studies reported that social media monitoring allowed for the detection of black box warnings, labeling changes, or withdrawals 3 months to 9 years in advance [14-17,20-24]. Most (88.9%) of these studies were conducted on specialized health care social networks and forums (Figure 4). One post that mentioned the occurrence of benfluorex (Mediator)-cardiac valvulopathy in a woman was posted in a patient forum 7 months before withdrawal [20]. Chee et al [24] identified 4 drugs (hydromorphone [proprietary name: Palladone], cerivastatin [Baycol], trovafloxacin [Trovan], and rofecoxib [Vioxx]) that were withdrawn by analyzing Yahoo Groups. Furthermore, posts related to sibutramine, which was not a watchlist drug at the time of the study but was later withdrawn owing to the risk of cardiac arrest and stroke, were observed more than a year ago [24]. Sibutramine was the fifth-highest-risk drug in the study [24]. In Duh et al’s [14] study on sibutramine, social media mentions of sibutramine-related cardiac issues helped predict those in FAERS 11 months later. In a study by Adjeroh et al [21], 30 of 46 (65.2%) drugs were detectable prior to FDA alert. Depending on the drug, adverse events could be detected 3 months (fluvastatin [Lescol]) to 35 months (codeine and atorvastatin [Lipitor]) before the FDA alert [21]. Wu et al [22] reported that the discussion frequency of arrhythmia due to propoxyphene/acetaminophen (Darvocet obviously exceeded the threshold since 2006, 4 years before the drug’s recall. Adverse events of 3 other drugs, namely simvastatin (Zocor) drospirenone/ethinyl estradiol (Yaz and Yasmin), were also detected 4-6 years prior to FDA action [22]. Yang et al [15] and Yang et al [16] analyzed the same data by association rule mining and the tensor-based technique. Association rule mining detected 6 of 14 ADRs, which included fluoxetine (Prozac)–induced suicidal thoughts (1 year in advance), methylphenidate (Concerta)–induced blurred vision (2 years in advance), fluoxetine (Prozac)–induced depression (5 years in advance), fluvoxamine (Luvox)–induced suicidal thoughts (5 years in advance), simvastatin–induced kidney disease (6 years in advance), and lansoprazole-induced diarrhea (13 years in advance) [15]. The tensor-based technique allowed more ADRs (11 of 14) to be detected 1-7 years earlier than association rule mining [16]. Feldman et al [17] reported that unsupervised relation extraction from web-based forums identified statin-induced cognitive impairment (labeling changes in 2012) as early as 9 years prior to the FDA label change. In the same study, Feldman et al [17] identified a significant relationship between bupropion (Wellbutrin) and agitation 7 years before FDA action. AZDrugMiner, developed by Liu et al [23], could detect rosiglitazone (Avandia)–induced myocardial infarction (18% of adverse events) and cardiac disorder (13% of adverse events) at high frequency from patient forums.

Five (35.7%) studies reported modest or negative results. Of these, 2 (40%) used generic SNS, and 1 (20%) used generic SNS and specialized health care social networks and forums (Figure 4). Caster et al [12] reported that signal detection in social media performed poorly and was not recommended. The area under the receiver operating characteristic curve of social media varied between 0.47 and 0.53 for the reference sets, while that of VigiBase ranged between 0.64 and 0.69 [12]. On Twitter and Facebook, only 16% and 6% of positive controls were detected before their index dates in the WEB-RADR and Harpaz references, respectively [12]. In a study by Pierce et al [13], proto-AEs were observed for only 2 of 10 drugs studied. Dronedarone-induced vasculitis reported by the FDA in April 2012 was indicated on social media in December 2010. However, as FAERS received its first report in July 2010, social media was 5 months late [13]. Only 1 positive signal was identified on social media before FAERS. The correlation between Banana Boat sunscreen and skin burns was observed on social media on June 2, 2012, and the FAERS first report appeared 17 days later [13]. Coloma et al [18] reported that the number of posts on rosiglitazone-induced cardiovascular events tended to increase with regulatory action. However, only 10 of 2537 posts described personal experiences of rosiglitazone-induced cardiovascular events. Patki et al [19] classified posts with or without ADR and classified the drugs as black box or normal drugs on the basis of the number of ADR-related posts. The classifier for black box drugs revealed a modest F-score of 0.6 [19]. Levofloxacin (Levaquin) and baclofen were misclassified as normal drugs because the number of posts was small, and the posts mainly referred to usefulness and not ADRs [19].

### Challenges

Table 2 categorizes the study limitations. There are currently several challenges associated with the use of social media for pharmacovigilance in terms of social media coverage, data quality, and analytic processing.

**Table 2:** Challenges associated with the use of social media for pharmacovigilance.

Challenges			References
**Coverage**			
	Limited social media coverage (generalizability to other data sources)	[12-14,18]	
	Lack of user population representation	[18]	
	Not a balanced coverage of all drugs and medical conditions	[14,18,24]	
	Limited coverage period	[12,14]	
**Data quality**			
	Use of colloquial language: misspellings or use of nonmedical terms and slang	[13,15,16]	
	Data duplication (double-counting)	[13,14]	
	Lack of medical and demographic information	[14]	
	Lack of causality information	[25]	
	Nonvalidated or incomplete data or misinformation	[14]	
	Low signal-to-noise ratio	[12,14,18,19,25]	
**Processing**			
	Curation burden due to data volume	[13]	
	Word-level analysis or does not reach semantic or discourse levels	[15,18,22-24]	

Different types of social media platforms have different user characteristics and types of data. Since studies have been conducted on limited social media platforms, analytical methods that were applicable to one platform may not be suitable on another platform. Typically, younger individuals use social media; therefore, adverse events that occur mainly in older people or those associated with drugs used for geriatric diseases can be underestimated. Data obtained from patient forums tend to be biased toward specific patients or drugs.

The low signal-to-noise ratio of the data makes the preprocess and analytical process burdensome. Social media, of course, is noisier than databases that collect only adverse drug events. In particular, the number of posts on generic social networking sites that are unrelated to adverse events is much higher than that of specialized health care social networks and forums.

There are extensive social media data to classify manually, but an understanding of natural language has not yet reached human levels. Nonetheless, many studies analyzed whether drug and adverse event–related terms are in the same sentence or post.

## Discussion

### Principal Findings

As interest in the use of social media in pharmacovigilance has increased, there have been steadily review papers on pharmacovigilance using social media. Most of the reviews focused on the data sources or analytical methods used in individual studies. Positive controls have allowed us to assess whether social media can detect significant unknown signals. A recent review [27] summarized studies that determined whether signals can be detected through social media before the actions of regulatory authorities. In this scoping review, we reviewed 14 studies on the application of social media in detecting new black box warnings, labeling changes, or withdrawals. Some studies in our review are not included in other previous reviews such as the one by Caster et al, who expressed a negative opinion. Our scoping review has the advantage of being the most state-of-the-art review that analyzed detection performance (eg, time interval between signals detected on social media and regulatory authority action).

Most studies have reported that meaningful signals could be detected before regulatory authorities take action. Signals were identified 3 months to 9 years in advance on social media, depending on social media sources and drug-ADR pairs. Most studies that have reported the usefulness of social media have used specialized health care social networks and forums. These sources have more patient experiences while fewer unrelated posts, which have helped overcome the disadvantages of low signal-to-noise ratios. Therefore, specialized health care social networks and forums are suitable sources for pharmacovigilance using social media. Furthermore, drugs such as benfluorex, sibutramine, drospirenone/ethinyl estradiol, methylphenidate, and fluoxetine, which may interest social media users, have yielded favorable outcomes.

A recent study by Caster et al [12], which used a large number of social media platforms, revealed a poor performance. Study drugs were not actively included on social media in their study. The detection performance of social media varies depending on the study drug, and social media–based pharmacovigilance will be useful for restrictive drugs. Thus, an approach to select drugs to monitor adverse events with social media will need to be developed. Differences in reference sets and statistical analysis also explain why Caster et al’s findings differed from those of other studies. The study used the WEB-RADR reference (independent of regulatory action) as well as the Harpaz reference (based on FDA labeling changes). Disproportionality analysis, which is commonly used for signal detection in spontaneous adverse event–reporting systems, was used instead of other methods tailored to the analysis of social media. The results of a pilot study conducted in 2018 are in line with those of the study by Caster et al [12]. Studies have reported 6 proto-AEs on Twitter and patient forums that meet the criteria for disproportionality [7]. Five of 6 selected adverse events were observed a median of 252 (range 144-367) days later on social media [7]. Negative views in recent studies have made the application of social media hesitant. We eagerly anticipate the results of the Adverse Drug Reactions from Patient Reports in Social Media study, which is currently underway in France [28].

Several challenges are associated with the use of social media for pharmacovigilance. Studies have reported a coverage problem, which makes their findings difficult to generalize. Therefore, pharmacovigilance cannot be entirely dependent on social media, but rather social media can be used to supplement existing pharmacovigilance measures. The challenges of low signal-to-noise ratios and word-level analysis might be resolved by enhancing natural language processing (NLP) and machine learning algorithms. While there have been recent rapid advances, computers still cannot fully understand human language at a semantic or discourse level. NLP techniques will need further improvements to meaningfully analyze the vast amount of RWD in social media.

### Limitations

This study has several limitations. First, while there was no restriction on the language of the papers retrieved from PubMed and EMBASE and references to related papers were searched, there may still be studies that we missed. Second, social media platforms that were widely used or derived from previous reviews were used as search terms. Therefore, we may have possibly missed information on minor social media platforms. Lastly, this is a rapidly developing field. Although we updated our search before submission, new studies may be published as of this publication. In particular, the Adverse Drug Reactions from Patient Reports in Social Media study is ongoing; hence, our results will need to be updated.

### Conclusions

Social media, along with conventional pharmacovigilance measures, can be used to detect signals associated with new black box warnings, labeling changes, or withdrawals. Several challenges remain; however, social media will be useful for signal detection for frequently mentioned drugs in specialized health care social networks and forums. Further studies are required to advance NLP and mine RWD on social media.

## Appendix

Multimedia Appendix 1 Supplementary Tables. Table 1: Search terms. Table 2: Analytical methods.

## Abbreviations

ADR: adverse drug reaction
FAERS: FDA Adverse Event Reporting System
FDA: Food and Drug Administration
NLP: natural language processing
Proto-AEs: posts that resembled adverse events
RWD: real-world data
SNS: social networking site
