# Supplementary material for: The Use of Social Media in Detecting Drug Safety–Related New Black Box Warnings, Labeling Changes, or Withdrawals: Scoping Review
Source: JMIR Public Health Surveill. 2021 Jun 28;7(6):e30137. doi: 10.2196/30137 (PMC8277336; doi:10.2196/30137)
Supplement: Multimedia Appendix 1 [file publichealth_v7i6e30137_app1.docx]

**Table 1.** Search terms

| PubMed | (social media[mesh] OR social networking[mesh] OR social medi*[tiab] OR social network*[tiab] OR blogging[mesh] OR blog*[tiab] OR Crowdsourcing[mesh] OR crowd sourc*[tiab] OR crowdsourc*[tiab] OR ((patient*[tiab] OR discussion[tiab] OR web[tiab] OR chat[tiab] OR internet[tiab] OR online[tiab]) AND (forum*[tiab] OR fora[tiab] OR message board*[tiab])) OR online communit*[tiab] OR Facebook[tiab] OR YouTube[tiab] OR Whatsapp[tiab] OR Weixin[tiab] OR wechat[tiab] OR instagram[tiab] OR tiktok[tiab] OR qq[tiab] OR sina weibo[tiab] OR qzone[tiab] OR reddit[tiab] OR kuaishou[tiab] OR snapchat[tiab] OR pinterest[tiab] OR twitter[tiab] OR medhelp[tiab] OR dailystrength[tiab] OR webmd[tiab] OR breastcancer.org[tiab] OR revolutionhealth[tiab] OR healthboards.com[tiab]) AND (Product Surveillance, Postmarketing[Mesh] OR Drug-Related Side Effects and Adverse Reactions [Mesh] OR Pharmacovigilance [Mesh] OR side effect*[tiab] OR adverse effect*[tiab] OR adverse reaction*[tiab] OR adverse event*[tiab] OR toxic*[tiab] OR pharmacovigilance[tiab] OR postmarketing surveillance[tiab] OR post-marketing surveillance[tiab] OR pharmacoepidemiology[mesh]) AND (blackbox OR boxed OR black box OR warning* OR withdraw* OR alert*) |
| --- | --- |
| EMBASE | #1 social media'/exp  #2 social medi*':ab,ti  #3 social network'/exp  #4 social network*':ab,ti  #5 blogging'/exp  #6 blog*:ab,ti  #7 crowdsourcing'/exp  #8 crowdsourc*:ab,ti  #9 crowd sourc*':ab,ti  #10 Facebook:ab,ti OR YouTube:ab,ti OR Whatsapp:ab,ti OR Weixin:ab,ti OR wechat:ab,ti OR instagram:ab,ti OR tiktok:ab,ti OR qq:ab,ti OR sina weibo:ab,ti OR qzone:ab,ti OR reddit:ab,ti OR kuaishou:ab,ti OR snapchat:ab,ti OR pinterest:ab,ti OR twitter:ab,ti OR medhelp:ab,ti OR dailystrength:ab,ti OR webmd:ab,ti OR breastcancer.org:ab,ti OR revolutionhealth:ab,ti OR healthboards.com:ab,ti  #11 ((patient* OR discussion OR web OR chat OR internet OR online) NEAR/3 (forum* OR fora OR 'message board*')):ab,ti  #12 online communit*':ab,ti  #13 1 OR 2 OR 3 OR 4 OR 5 OR 6 OR 7 OR 8 OR 9 OR 10 OR 11 OR 12  #14 postmarketing surveillance'/exp  #15 adverse event'/exp  #16 side effect'/exp  #17 pharmacovigilance'/exp  #18 pharmacoepidemiology'/exp  #19 ('side effect*' OR 'adverse effect*' OR 'adverse reaction*' OR 'adverse event*' OR toxic*):ab,ti  #20 (pharmacovigilance OR 'postmarketing surveillance' OR 'post-marketing surveillance'):ab,ti  #21 14 OR 15 OR 16 OR 17 OR 18 OR 19 OR 20  #22 blackbox OR boxed OR 'black box'  #23 warning*  #24 withdraw*  #25 alert*  #26 #22 OR #23 OR #24 #25  #27 #13 AND #21 AND #26 |

**Table 2.** Analytical methods

| Number | Author | Analytic methods |
| --- | --- | --- |
| 1 | Caster et al. (2018) [12] | Disproportionality analysis: four signal detection algorithms using IC_025_ >0, (PRR >2 and N ≥3), (PRR >2 and N ≥3 and χ^2^ ≥4), and (PRR_025_ >1 and N ≥3)  Performance evaluation using ROC  Comparison with VigiBase |
| 2 | Pierce et al. (2017) [13] | Machine learning algorithms: Automated document classification that categorized each post as either a Proto-AE or a non-Proto-AE  Manual annotation: Posts with indicator scores of at least 0.65 were reviewed by a team of human curators  An independent third-party medical reviewer conducted causality assessments |
| 3 | Duh et al. (2016) [14] | Manual annotation  Time series analysis  Comparison with FAERS |
| 4 | Yang et al. (2015) [15] | Resorted to CHV to build up ADR lexicon  Association rules mining: calculating confidence, leverage, and lift  Temporal dimension: each year’s dataset is considered as a process unit to detect some strong signals that may only occur during a certain time interval |
| 5 | Yang et al. (2015) [16] | Matrix-based technique: same as those presented in literature number 4. Only one example of the analysis results is given, and the detailed results are given in the literature number 4.  Tensor-based technique: exploring the multi-dimensional structure (drug × ADR × time) by using CANDECOMP/PARAFAC (CP) decomposition and CP weighted optimization  Comparison of matrix-based technique and tensor-based technique |
| 6 | Feldman et al. (2015) [17] | Utilizing unsupervised relation extraction framework and augmenting its lexicon with respect to the medical domain to mine drug-ADR relations  Calculating lift |
| 7 | Coloma et al. (2015) [18] | Manual annotation  Time series analysis |
| 8 | Patki et al. (2014) [19] | Two machine learning algorithms: multinomial naïve Bayes and support vector machines were used for the binary classification of comments into ADR and noADR. Features included N-grams, synset expansions, change phrases, and sentiword  Combining classification probabilities: compute the probability of each drug belonging to normal or black box, given the probabilities assigned to the comments by automatic classifiers |
| 9 | Abou Tamm et al. (2014) [20] | Manual annotation  Frequency analysis: before November 2009 (before withdrawal), between November 2009 and November 2010 (communication of risk of valvulopathy related to benfluorex in social media), and after November 2010 |
| 10 | Adjeroh et al. (2014) [21] | Peak-labeling fusion scheme: Multilevel cross-channel signal fusion using within-channel multiple temporal windows to combine the information derived from diverse sources |
| 11 | Wu et al. (2013) [22] | Discriminative classification: compute discussion frequency and compare it with threshold (the average frequency of known side effects and that of unknown side effects)  Generative modeling: estimate how likely the side effect is related to the drug |
| 12 | Liu et al. (2013) [23] | Medical entity extraction: applied multiple types of lexicon sources to extract drug names and adverse events from the text, including UMLS, FAERS, and CHV  Adverse event extraction: relation extraction and relation classification  Report source classifier: machine learning classifier to identify reports based on patient experience  Comparison with FAERS |
| 13 | Chee et al. (2011) [24] | Machine learning algorithms: feature vectors included general word features (5000, 10000, and 15000 features selected using Bi-Normal Separation including unigram, bigrams and trigrams) and specialized lexicons (drugs, medical terminology, sentiment, ADR, and diseases) |
| 14 | Chee et al. (2009) [25] | Sentiment analysis: Calculate the ratio of negative emotion words (Linguistic Inquiry and Word Count lexicon) to total words |

ADR, adverse drug reaction; CHV, Consumer Health Vocabulary; FAERS, Food and Drug Administration Adverse Event Reporting System; IC, Information Component; Proto-AEs, posts with resemblance to adverse events; PRR, Proportional Reporting Ratio; ROC, receiver operating characteristics; UMLS, Unified Medical Language System
